# Aquatic animal resources in Prehistoric Aegean, Greece

**DOI:** 10.1186/2241-5793-21-2

**Published:** 2014-05-13

**Authors:** Dimitra Mylona

**Affiliations:** Institute of Aegean Prehistory for East Crete, 59 E. Daskalaki, 74100 Rethymno, Greece

**Keywords:** Prehistoric fishing, Fish remains, Molluscan remains, Fish processing, Archaeology of fishing, Prehistoric aquatic resources

## Abstract

This paper explores the early stages in the history of fishing in the Aegean Sea in Greece, and highlights its formative phases and its specific characteristics in different points in time. This is testified by various physical remains, such as fish bones, fishing tools, and representations in art, which are gathered in the course of archaeological research. The aquatic resources in the Aegean Sea have been exploited and managed for millennia by communities that lived near the water and often made a living from it. The earliest evidence for a systematic, intensive exploitation of marine resources in the Aegean Sea dates to the Mesolithic, eleven millennia ago. In the Neolithic period, the adoption of a sedentary, agro-pastoral way of life led to a reduction in the intensity of fishing and shellfish gathering. Its importance as an economic resource remained high only in certain regions of rich, eutrophic waters. In the Bronze Age, an era of social complexity and centralized economy, the exploitation of aquatic, mostly marine, resources became a complex, multi-faceted activity which involved subsistence, industry and ideology. The range of preferred fish and invertebrate species, the fishing technology, and the processing of fish and shellfish in order to produce elaborate foods or prestige items are all traceable aspects of the complex relationship between humans and the aquatic resources throughout the prehistory of fishing and shellfish gathering in the Aegean area. The broadening of collaboration between archaeology and physical sciences offers new means to explore these issues in a more thorough and nuanced manner.

## Introduction

The aquatic resources of the Hellenic area have been systematically exploited by coastal communities that lived by the sea, the rivers and the lakes, for a very long period of time. This interaction begun at least as early as the 11th millennium BP (Before Present) and it lead to a wide range of fishing choices and strategies. In these one can trace adaptations to the local ecosystems but also a reflection of the interests and priorities of the fishing communities involved in the exploitation of these resources. Despite the observed variability there are certain constant features which survived through the millennia to the modern era. The range of fish and shellfish, fishing tools and processing methods are some of these features. This paper provides a short review of these issues in the context of prehistoric Aegean, a period in time when the basic features of the exploitation of aquatic resources were formulated.

Ancient fishing is explored through a multi-level approach by archaeology and history. The main categories of data for such an approach are the archaeological remains of aquatic resources and fishing tools as well as records in literature and representations in art. The aquatic animal remains, mostly fish bones, sea shells, crustaceans and coral skeletons, are identified using reference collections and relevant monographs; various features of these remains are recorded, particularly those that are pertinent to the animals’ exploitation by humans [1, 2]. Remains of fishing implements (most commonly their durable elements such as the bone or metal fishing hooks, the stone weights or the pumice floaters) are also recovered archaeologically [3]. Records of aquatic animals in ancient texts, along with relevant representations in art provide further evidence on fishing related matters. They also illustrate an elusive aspect of the past, i.e. how people thought and felt about the aquatic resources and their harvesting [4]. However, the exploitation of these resources and the particular choices made by the different communities in different times and locations, are governed not only by cultural rules and traditions but also by the restrictions imposed by the dynamics of the aquatic environments and the biology and ethology of the exploited organisms [5]. Therefore, archaeological and historical research is supplemented by a range of natural sciences, such as ichthyology, marine biology, chemistry, etc. It should be emphasized that such a combined approach, effective as it may be, does not provide a snapshot of the available aquatic resources at that period but it reflects the resources that were accessed by humans, of those elements that were used by people as food, raw material and/or symbols.

## Review

### The abundance of marine resources in the Mesolithic

The earliest evidence for the systematic, complex and precisely orchestrated exploitation of aquatic resources dates to the 11th millennium BP, at the end of an era of rapid environmental changes and the beginning of the Holocene. This period is conventionally called the Mesolithic. It is the era of the opening of the Black Sea to the Aegean Sea, which along with the increased flow of the large rivers in Northern Greece led to increased productivity of the Aegean Sea [6–8]. Culturally the Aegean shores were sparsely populated by communities of hunters, gatherers, and fishermen [9, 10]. There is unequivocal evidence that Mesolithic people, were able to cross considerable distances between the mainland and the Aegean islands of the time [11, 12]. Three archaeological sites, two caves (Franchthi Cave in Argolid [13] and the Cave of Cyclops at Yioura in Sporades [14, 15]) and the open air site of Maroulas on Kythnos [16] provide ample evidence for fishing during the early Holocene. Excavation at these sites produced thousands of fish bones and scales, as well as a large number of sea shells, marine mammal bones and sea bird bones. The ensuing discussion is based on data from a number of sites [17–29].

**Figure 1:**
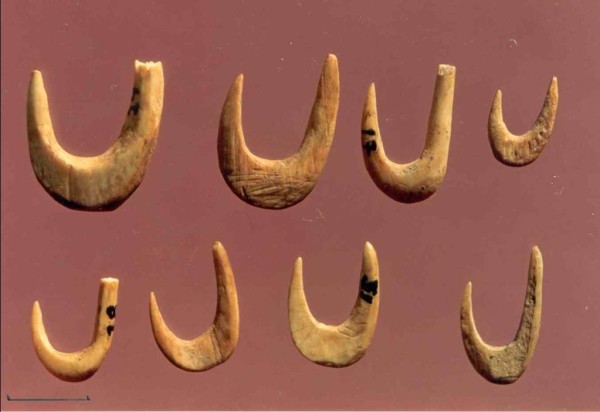
**Cave of Cyclope, Youra.** Bone fish hooks (A. Sampson).

The fish present at these sites were mostly inshore species of medium size (20–40 cm). Groupers (*Epinephelus* sp.), scorpion fishes (Scorpaenidae), wrasses (Labridae) and also the John dory (*Zeus faber*), common dentex (*Dentex dentex*), common pandora (*Pagellus erythrinus*), white sea bream (*Diplodus sargus*) and other members of the Sparidae were the most common. The excavation in the Cave of Cyclops at Yioura revealed a part of the Mesolithic fishing technology that targeted these fish species, in the form of fishing hooks [30]. They are made of bone and antler and they come in two shapes, the bi-pointed gorges and the classic curved shape that survives to date (Figure 1). A second category of fish found in these sites are the euryaline species, with mullets (Mugilidae) being the most common. Their catch in considerable numbers can be taken as an indication for the presence of coastal brackish environments. This can only be verified for Franchthi, where special studies on the coastal morphology through time have been performed [20, 31]. What is particularly interesting, however, is the fact that even at Franchti, Mugilidae were never very common, or at least not as common as another gregarious type of fish, the migratory Scombridae.

**Figure 2:**
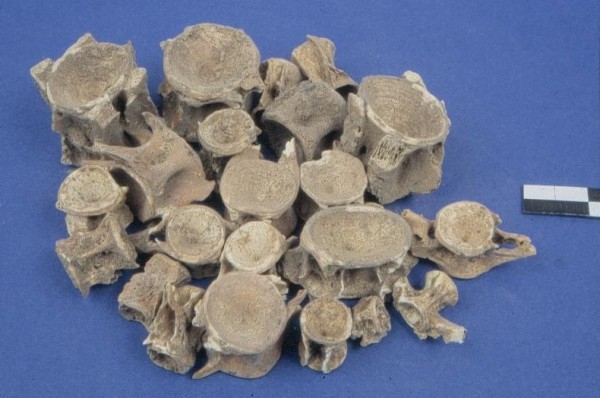
**Cave of Cyclope, Youra.** *Thunnus* sp. and Mugilidae spp. vertebrae (A. Sampson).

Remains of migratory fish are fairly common in all three Mesolithic sites mentioned above (Figure 2). Even though large tunas (*Thunnus* sp.) were regularly caught and consumed, with a preference towards small or medium pelagic individuals, fishermen mostly targeted the smaller species within the family (e.g. *Scomber japonicus*, *Euthynnus alletteratus, Sarda sarda* and *Auxis rochei*). This selectivity towards smaller sizes might be related to the ease by which smaller fish could be handled, as opposed to larger and heavier individuals.

**Figure 3:**
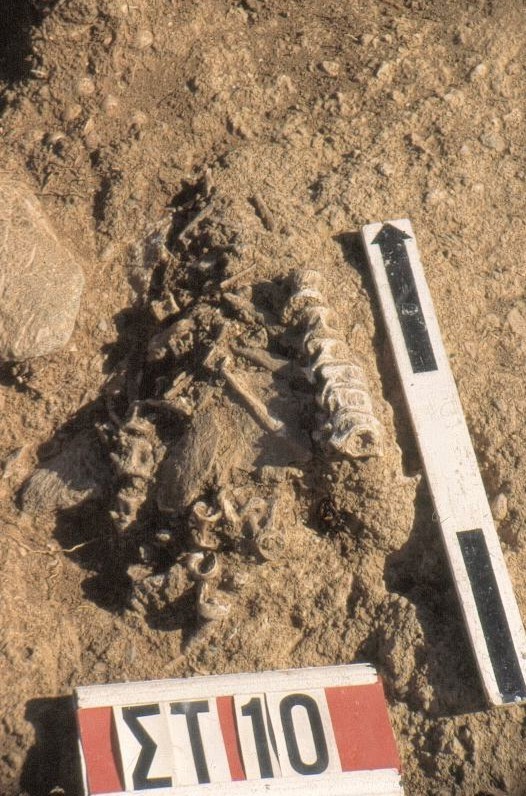
**Maroulas, Kythnos.** Two fish skeletons found in the floor of a circular hut (A. Sampson).

In the Mesolithic, the first evidence for fish processing was found. In the osteological assemblage from the Cave of Cyclops on Yioura, certain anatomical parts of some migratory fish, such as the first vertebrae and the cranial bones of little tunnies (*Euthynnus alletteratus*), are systematically under-represented or missing altogether. This fact suggests that these elements had been removed before the fish were brought in the cave. Traditionally, the removal of the head and innards is the first step in the process of fish preservation, especially for blood-rich fish such as the Scombridae. An interest in fish processing is also vividly illustrated in the open air settlement at Maroulas on Kythnos. At this site, the whole fish had been stored in the floors of the circular huts (Figure 3). As in Yioura, in some cases, the head bones and the first vertebrae were absent. No information on fish processing has been reported from Franchthi cave so far, but this is probably only because the analysis and subsequent publication of the results on fish remains from this cave is still ongoing. Evidence from other Mesolithic sites on Cyprus [32] but also in Southern Italy [33] suggest that already at that time a common fish preservation tradition had been developed in Central and Eastern Mediterranean.

### Fishing in lakes, rivers, and the sea in the Neolithic

The Mesolithic fishing bonanza, when marine aquatic resources were abundant and intensively exploited in coastal and near-coastal sites did not seem to continue in the following millennia. From the Neolithic period, between the 7th and the 4th millenium BC, after the adoption of agriculture and animal husbandry as the main economic modes throughout the Hellenic peninsula [34–37], the exploitation of aquatic resources, mostly the marine ones, diminishes. The contribution of fish and aquatic molluscs to the Neolithic diet never superseded that of the domestic animals (i.e. the cattle, the pig and the ovicaprids).

**Figure 4:**
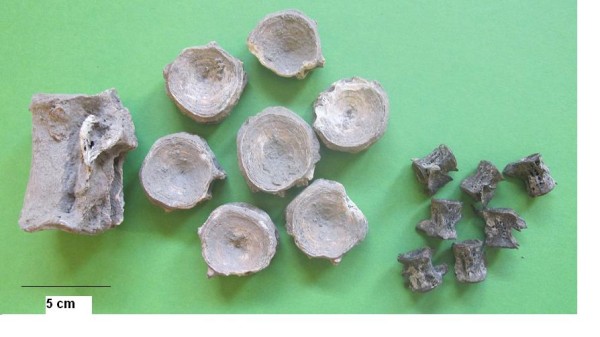
**Kryoneri, Serres.** Fish bones of *Silurus glanis* and Mugilidae spp. (D. Mylona).

At certain locations, however, especially in eutrophic areas, fishing and shell gathering remained an important activity, and some of the technological knowledge of the Mesolithic survived. The Neolithic lake-side settlement at Dispilio, on the south coast of the Lake Orestias near Kastoria, is one such example. To judge by their quantity, fish and mollusks were procured regularly and in considerable amounts and bone and antler fish hooks were also very common [38–41]. In the area of Amphipolis, near the estuaries of Strymon River in northern Greece, Neolithic people were making use of all three ecosystems, the river, the estuaries, and the sea. As a result, the remains of fish from all these habitats were found with emphasis, however, to those from the river and its estuaries. Various species of the Mugilidae family are particularly common at Kryoneri as are the various river fish such as the European perch (*Perca fluviatis*), the Northern pike (*Esox lucius*), the tench (*Tinca tinca*), and the common carp (*Cyprinus carpio*) and especially the eel (*Anguilla anguilla*) and the large cat-fish (*Silurus glanis*) (Figure 4), both of which acquired a fame of excellence in later Classical Greece [42]. Fishing and shellfish gathering was equally intensive in several other riverside or lakeside settlements in northern Greece, e.g. along the route of Aliakmon river or on the shores of now-dried up lakes such as the Giannista lake [43], but also at some coastal and insular locations, such as Agios Petros in Sporades [44] or Makrygialos on the coast of Pieria [45] and others [46]. In the Neolithic era we have robust evidence that shellfish gathering was not only diet-related but served other purposes as well. *Spondylus* shells, for example, that were gathered in the Aegean Sea, were modified into ring-shaped ornaments and, through a complex exchange network, they travelled to Central Europe [47–49].

### Bronze Age exploitation of the aquatic resources as a multi-level act

**Figure 5:**
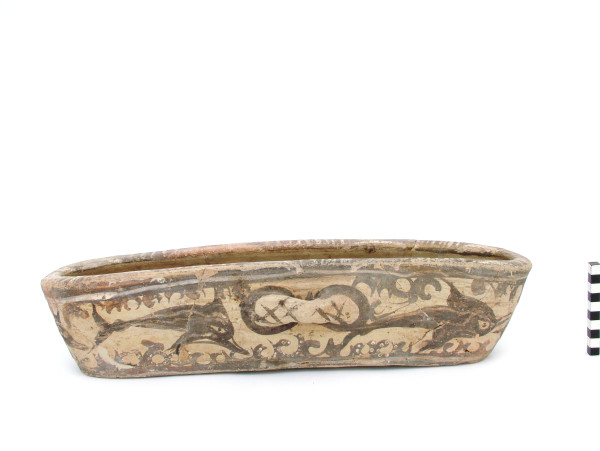
**Akrotiri, Thera.** Open vessel decorated with dolphins and marine vegetation (Akrotiri Excavations Archive).

In the Bronze Age (3rd and 2nd millennium BC), our understanding of fishing and fishing products increases exponentially. The picture drawn by archaeology is both complex and detailed. The character of fishing in the Aegean Sea, as far as the exploited species and the relevant fishing technology is concerned, was consolidated. Aquatic, mainly marine, organisms were systematically processed on a large scale not only for food but also for the production of luxury products. Marine elements, physical and manmade such as octopus, fish, shellfish of various kinds, marine vegetation, and rocks, as well as ship of various types, naturalistic or more schematic, became popular decorative motifs in art [50, 51] (Figure 5). More clearly than before, in this period, the sea and the aquatic animals participated in the social and religious ritual [52].

**Figure 6:**
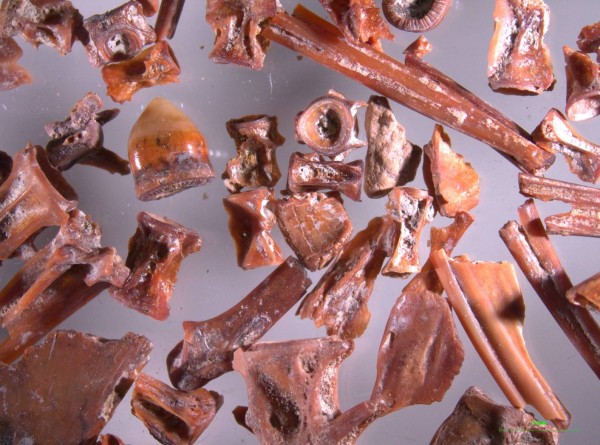
**Akrotiri, Thera.** Fish bones of small inshore fish (Akrotiri Excavations Archive ).

In the Bronze Age most of the consumed fish throughout the Aegean Sea were inshore fish of the shallow or even very shallow waters (Figure 6). The following discussion is based on data from various sites: Palaikastro [53], Mochlos [54, 55], Pseira [54, 56], Kommos [54, 57]. In southern Aegean Sea, the picarels (Centracanthidae) and the bogues (*Boops boops*), were most commonly caught species, while the annular sea bream (*Diplodus annularis*), the comber (*Serranus cabrilla*), the damsel fish (*Chromis chromis*), the small individuals of common pandora (*Pagellus erythrinus*), and the parrot fish (*Sparisoma cretense*) followed. But even larger size fish, such as the red porgy (*Pagrus pagrus*), the scorpion fish (Scorpaenidae), the leer fish (*Lichia amia*), the European barracuda (*Sphyraena sphyraena*), the stingray (*Dasyatis* sp.), and the sharks are fairly common. In other words, at least in the southern Aegean Sea, fishing was done in the shallows, very near the coast.

The migratory fish, which seasonally approach the coast and are traditionally caught by stationary traps that are linked to the coast [4], are less often caught in the southern Aegean than in the north. They are not altogether an untapped resource however. The wall paintings of the “Little Fisherman” at Akrotiri, vividly illustrate the capture of dolphin fish (*Corypahena hypurus*) and of little tunnies (*Euthynus aleteratus*) or bullet tunnies (*Auxis rochei*) [58, 59]. At the same site, a second piece of evidence suggests that the migratory fish were exploited even if not in large scale; this is the unique finding of two slices of tuna (*Thunnus* sp.) discovered in a frying pan. These were cooked in a makeshift kitchen, probably just hours before the catastrophic volcanic eruption occurred [60].

**Figure 7:**
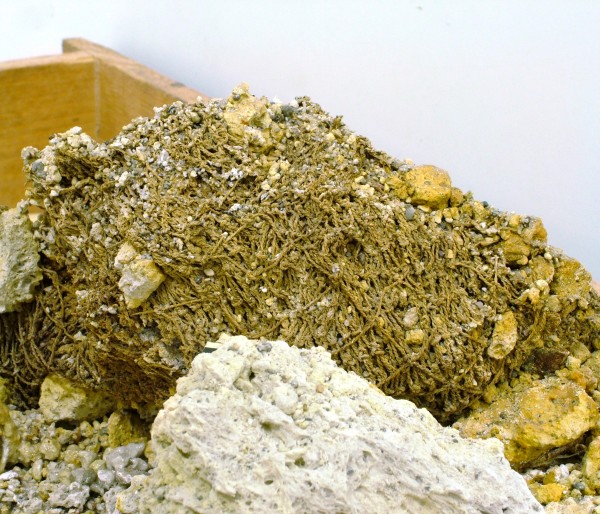
**Akrotiri Thera.** Fishing net (Akrotiri Excavations Archive).

The fishing tools used in this period are adapted to the inshore fishing zone. Bronze hooks were widely used and we find them in all sizes and configurations, simple or complex, with a barb or without [3], some having a closed shape for the capture of bottom feeders and other having a more open shape for surface swimmers. Nets were also widely used, and we do find a variety of types, simple nets, trammel nets, and cast nets being the most common [3]. The nets are only rarely preserved archaeologically, due to the perishable nature of their fibers. Archaeological sites with good preservation, such as Akrotiri on Thera, provide such examples (Figure 7) [61]. What is usually preserved are the non-perishable, metal or stone elements of the nets, such as the lead folded sheets or the perforated pebbles, both of which functioned as weights [3, 62]. There is even some evidence, that in the Bronze Age, baited baskets and the stationary fish traps, which at later periods are known as *thynneia*, were in use.

**Figure 8:**
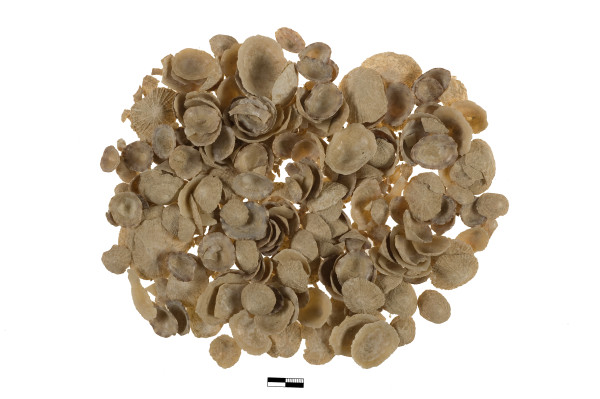
**Papadiokambos, Siteia.** Limpet shells (*Patella* sp.) (Ch. Papanikolopoulos – Papadiokambos Excavations Archive).

The gathering of edible shellfish and crustaceans follows the same motif (Figure 8). The top shells (*Monodonta* sp.), the limpets (*Patella* sp.) and the crabs were apparently consumed systematically, and at places in very large quantities. The ensuing discussion is based on data from the following sites: Palaikastro [63], Papadiokambos [64], unpublished observations, Mochlos [65], Pseira [66–68], and Kommos [69–71]. These animals are found in the mediolittoral zone, and can be gathered with hardly any technological investment and even minimal dexterity. This pattern is also broadly applicable in northern Greece, despite the fact that rich molluscan resources from different habitats, such as river estuaries and coastal lagoons were available and were exploited to some degree [46].

**Figure 9:**
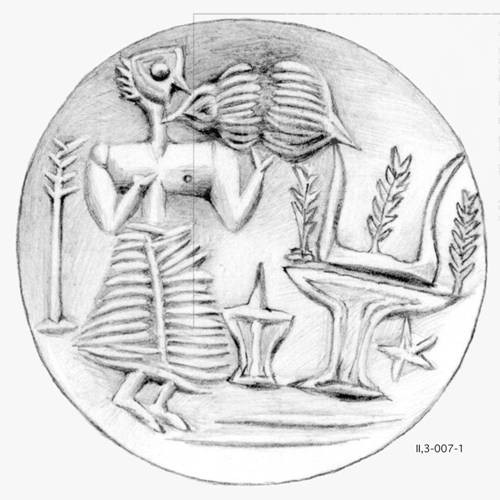
**Idaeon Andron, Rethymno.** Ritual scene with a triton (*Charonia* sp.) shell in use (CMS-II, 3-007-1).

Shellfish gathering for special purposes other than the culinary was fairly different. It targeted species of deeper waters and it required diving skills and/or specialised technology both for the capture and processing of these animals. The fan mussel (*Pinna nobilis*), the purple shellfish (Muricidae) and the tritons (*Charonia* sp.) are some such species. The fun mussels were used for the production of decorative iridescent inlays [72]. The tritons were used modified or in their natural state as vessels for transferring liquids, as ceremonial vessels and as musical instruments (Figure 9) [73]. Large quantities of purple shellfish were used for the systematic production of purple dye on an industrial scale, from as early as 1800 BC [74].

**Figure 10:**
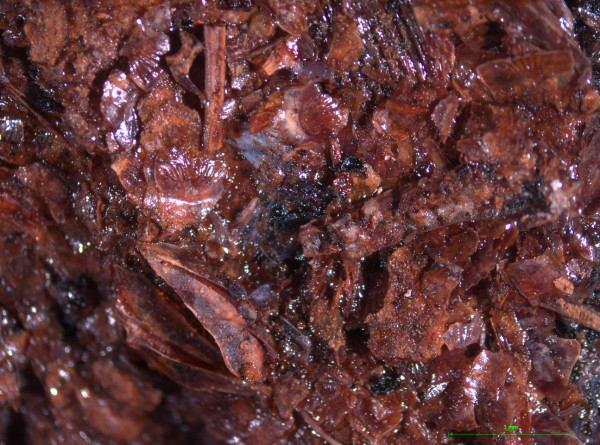
**Akrotiri, Thera.** Detail of the fish paste some fish bones can be discerned (D. Mylona).

Similarly, special, articulated technologies developed around fish processing. The best examples for this were revealed by the excavations at the Bronze Age site of Akrotiri on Thera [75]. A massive volcanic eruption at around 1650 BC covered the affluent, urban settlement of Akrotiri with a thick layer of volcanic ash. This material preserved the remains of the town in pristine condition. As a result, the archaeological excavations reveal a wide range of organic remains, which elsewhere would have disintegrated over time. Recent excavations revealed not only the fragile fishing net mentioned earlier but also another unique finding: a small storage vessel contained the desiccated remains of what appears to be a fish paste (Figure 10). This was made using various small fish such as picarels or bogues and small sting rays, seeds of an unidentified type of cereal and possibly other condiments, which have not been identified yet. This type of preserved fish product was known in more recent times, in the Roman world, as *alec* and was produced by fermentation of small fish and the innards of larger ones with the addition of salt, vinegar and other ingredients [76].

**Figure 11:**
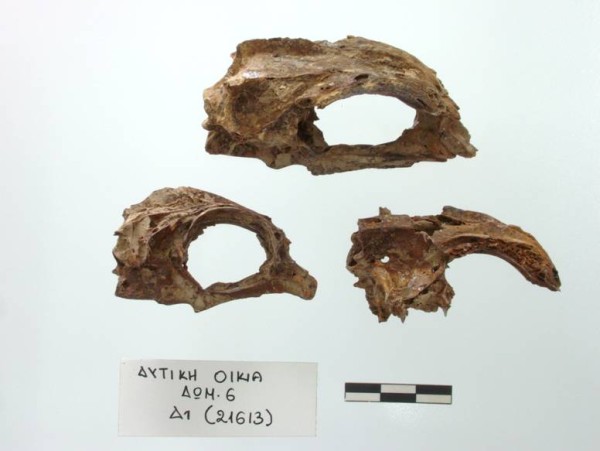
**Akrotiri, Thera.** Articulated crania of *Pagrus pagrus* found in a pithos (Akrotiri Excavations Archive).

At the same location the excavators found the remains of several large-sized common dentex (*Dentex dentex*) individuals. The retrieved remains were articulated, and even a whole preserved fish, bones and flesh, was found. It is interesting that among these fish bones only the first and the last vertebrae were present, while the rest were missing altogether. It appears that the fish had been opened along their length, their vertebral column removed and the fish were probably salted and/or dried and hung on a string. Akrotiri provided another example of a different fish product, which was found in the ground floor store room of the so called “West House”, the building which was decorated with the wall-paintings of the “Little Fisherman” mentioned earlier. A storage vessel contained the remains of a large number of red porgy (*Pagrus pagrus*) of similar size (Figure 11) and several seeds of an unidentified type of cereal. It appears that whole fish had been preserved in this vessel, making up the third identified type of a fish processing product at Akrotiri.

The special uses and the technologies involved in the exploitation of the marine molluscs and the elaborate processing of fish in the Bronze Age placed the sea and its creatures into spheres other than the dietary and the technological. These are the spheres of social competition and ideology.

## Conclusions

The research on the exploitation of aquatic resources in antiquity was vastly enriched in the last decades by the collaboration of archaeology with biology and ecology. This rendered the physical remains of aquatic organisms, such as fish bones, sea shells, etc., eloquent testimonies of past fishing practices. Recent scientific developments open up more possibilities for collaboration between the archaeology of aquatic resources and the natural sciences. Molecular genetic analyses for identifying the remains of aquatic animals or their by-products e.g. [77, 78] and isotopic analysis e.g. [48] for exploring issues of provenance, diet, etc., are two such examples.

The exploration of the character of fishing and fishing products in the distant past reveals a picture which is both familiar and exotic. The sea, its organisms, the fishing tools and methods, the processing and consumption of aquatic foods are all very similar to what is known from Greece of the previous decades. The societies involved in fishing and consuming its products, however, were different on many aspects. A plethora of evidence suggests that the meanings given to these familiar activities were also different in those societies. Today, in this era of globalization, the relationship between the “common” and “familiar” on the one hand and the “different” and “strange” on the other, as these emerge from the study of fishing in the past, is particularly relevant.

## Authors’ information

DM is an archaeologist, who specializes in zoo-archaeology, with special emphasis in the analysis of remains of aquatic animals. She got her doctoral degree in Archaeology, University of Southampton (UK), and she wrote a thesis entitled “Fish-eating in Greece from the fifth century BC to the seventh century AD: a story of impoverished fisherman or luxurious fish banquets?”. Her research focuses on various aspects of maritime communities that lived around the Aegean and the Mediterranean Sea in the past. DM is currently holding a post-doctoral position at the Institute of Aegean Prehistory, Crete, analyzing assemblages of animal remains from archaeological sites that range in date from Mesolithic to Byzantine.
